# Multi-Omics Integration in Nephrology: Advances, Challenges, and Future Directions

**DOI:** 10.1016/j.semnephrol.2025.151584

**Published:** 2025-04-11

**Authors:** Afaf Saliba, Yuheng Du, Tianqing Feng, Lana Garmire

**Affiliations:** * Center for Precision Medicine, Department of Medicine, University of Texas Health Science Center at San Antonio; † Department of Computational Medicine and Bioinformatics, University of Michigan Medical School

**Keywords:** Biomarker discovery, computational methods, data challenges, kidney disease, multi-omics integration

## Abstract

Omics technologies have transformed nephrology, providing deep insights into molecular mechanisms of kidney disease and enabling more precise diagnostic tools, therapeutic strategies, and prognostic markers. Multi-omics data integration, spanning bulk, single-cell, and spatial omics, offers a comprehensive view of kidney biology in health and disease. In this review, we explore methods and challenges for integrating transcriptomic, epigenomic, and spatial data. By combining omics layers, researchers can uncover novel molecular interactions and spatial tissue organization, advancing our understanding of diseases like diabetic kidney disease and autosomal polycystic kidney disease. This integrated approach is reshaping diagnostic and therapeutic strategies in nephrology and is critical for optimizing insights available from spatial and multi-omics analysis.

## INTRODUCTION

Recent advances in large-scale data generation and computational analysis have significantly impacted biomedical research and clinical medicine. While fields like cancer research have led precision medicine in these “big science” initiatives, other fields have lagged behind. However, recent advances hold exciting potential for nephrology to take the lead.^1^ In the era of big data, the advent of high-throughput omics platforms has markedly increased the volume of molecular data, encompassing the genome, epigenome, transcriptome, proteome, and metabolome, allowing a comprehensive understanding of human health and disease development through multimodal representation.^2^ Adopting an integrative approach to analyze multiple datasets is essential to address biological problems holistically.

Multi-omics integrative analysis can be applied to different data resolutions for various downstream tasks. For bulk omics, such integration typically aids in patient classification, disease subtyping, and the derivation of biological insights.^3^ Single-cell data integration offers a finer resolution, enabling the identification of cell subpopulations, marker genes, and comparative analyses that account for tissue heterogeneity.^4^ Furthermore, spatial data integrates the spatial context on top of single-cell (or spot-level) data, allowing the identification of spatial functional domains, capturing cell–cell interplay, and developing deeper insights into cellular and tissue constitution.^5^ Accompanying such complex data, multi-omics integration is fraught with challenges ranging from data heterogeneity and dimensionality to interpretability and validation.^6^ This review summarizes key recent advancements in omics data integration applied in nephrology while highlighting the major challenges, possible solutions, and future perspectives.

## OVERVIEW OF MULTI-OMICS TECHNOLOGIES IN NEPHROLOGY

Genomics, the study of an organism’s set of genes, has been instrumental in identifying genetic variants linked to kidney diseases through techniques like whole genome sequencing (WGS) and genome-wide association studies (GWAS). The WGS method identifies DNA variations beyond the exons, particularly in introns and noncoding regions, which can cause genetic disorders. These variations are missed by whole exome sequencing, which examines exons. Additionally, WGS detects complex trait variants, such as copy number variants and those in genes located within intronic and other noncoding areas of the genome. The readers interested in more details are referred to the reviews of Zoccali et al. and Devarajan et al.^7,8^ GWAS identifies single nucleotide polymorphisms among participants and is used to examine the DNA of large groups of individuals in order to detect genetic variations linked to specific diseases. GWAS uncovered specific genetic variants linked to chronic kidney disease (CKD), such as those in the shroom family member 3 (*SHROOM3*) gene, which have been shown to contribute to kidney fibrosis.^9,10^ A study by Li et al. combined GWAS with epigenomic and transcriptomic resources and identified 41 genome-wide significant loci related to estimated glomerular filtration rate in individuals of African ancestry and those with ancestry in the Americas. This approach not only revealed two novel loci but also highlighted potential effector genes and key regulatory elements involved in renal function and disease, emphasizing the value of diverse, omics data for advancing understanding and improving clinical outcomes in CKD.^11^ A limitation of GWAS is that single nucleotide polymorphisms may not directly explain changes in gene expression.

High-throughput gene expression measurements, including RNA sequencing (RNA-seq) and single-cell transcriptomics, have revolutionized nephrology by providing unprecedented insights into renal cell types and gene expression patterns. Kidney biopsy samples, fundamental to nephrology research, are a rich data source for deciphering disease etiologies and can now be used to reveal potential mechanisms with multi-omics data. These samples can be analyzed through bulk RNA-seq at various levels, including compartment specific and nephron segment specific, or via single-cell RNA-seq (scRNA-seq) to produce detailed transcriptomic profiles (reviewed in detail by Hill et al.^12^). RNA-seq has deepened our understanding of kidney fibrosis,^13^ identifying key cell populations such as myofibroblasts, which originate from pericytes and fibroblasts, contributing to extracellular matrix deposition in CKD. Moreover, scRNA-seq has further clarified distinct fibroblast subtypes and injury-associated proximal tubule cells, underscoring their critical roles in fibrosis progression and revealing new therapeutic targets such as targeting profibrotic signaling pathways and ferroptosis.^14^ Additionally, single-cell transcriptomics has greatly expanded our knowledge of kidney cellular diversity, including the discovery of unique podocyte subpopulations in focal segmental glomerulosclerosis, which enhances our understanding of disease mechanisms and supports personalized treatment approaches.^15^ A limitation of RNA-seq data is in RNA levels not always correlating with protein abundance.

Beyond transcriptomics, epigenetics and proteomics data can also enrich our comprehension of kidney biology. Epigenomics, which is the study of heritable changes in gene expression that do not involve alterations to the underlying DNA sequence, advanced our understanding of epigenetic modifications roles in kidney development, function, and disease. For example, increased DNA methylation in the Wnt/beta-catenin pathway was linked to age-related kidney dysfunction, and DNA methyltransferase 1-mediated methylation was critical for nephron progenitor maturation, preventing developmental defects by silencing transposable elements in the kidney.^16^ Another study examined cytosine methylation and open chromatin states in kidney samples from 399 individuals, including both CKD patients and controls, and discovered that significant methylation changes associated with kidney disease were influenced by genetic variants linked to the condition. Additionally, the study identified regions with both methylation and chromatin alterations that affected gene expression, especially in metabolism-related genes. Methylation risk scores were found to enhance disease classification and prediction, indicating a potential causal relationship between epigenetic changes and kidney disease, suggesting new possibilities for risk stratification.^17^

Moreover, the use of advanced mass spectrometry techniques, often coupled with liquid chromatography, has allowed for a detailed analysis of the kidney proteome, leading to the identification of disease-specific proteins such as the M-type phospholipase A2 receptor in membranous nephropathy, which has profoundly influenced diagnostic and therapeutic approaches in kidney disease.^18^

Metabolomics, unlike the other omics mentioned earlier, uses mass spectrometry combined with liquid chromatography to profile metabolites. A key distinction is that metabolites reflect the functional output of enzymatic pathways, indicating which pathways are active or suppressed under specific conditions compared to control conditions. This technique has uncovered the significant role of gut-derived compounds like indoxyl sulfate and p-cresol sulfate in the progression of CKD, thereby providing crucial insights into the metabolic disturbances associated with renal dysfunction.^19^ Another urine metabolomics study revealed 13 metabolites correlating with mitochondrial metabolism in diabetic kidney disease (DKD), with further studies assessing their roles in predicting future kidney function decline and understanding kidney disease progression.^20,21^ Also, acute kidney injury (AKI) metabolomics has revealed that niacinamide, a precursor in the salvage pathway of nicotinamide adenine dinucleotide (an essential cofactor for energy metabolism and cellular health), plays a significant role in protecting kidney function.^22^

While these methods have advanced our knowledge, they also have limitations, such as lack of single-cell resolution or spatial information. To address these challenges, advancements like spatial omics combining histologic imaging with spatial profiling and single-cell genomics can reveal the intricate tissue microenvironments and their functions.^23,24^ For instance, a recent study by Sharma et al.^24^ has identified endogenous adenine as a novel biomarker for unraveling the biological mechanisms behind DKD progression, using a combination of urine metabolomics, single-cell transcriptomics, and spatial metabolomics. Additionally, machine learning can analyze high-dimensional pathology data from digital biopsy images to reveal significant patterns.^25^

Beyond individual omics, multi-omics studies are crucial for identifying biomarkers and understanding disease mechanisms in nephrology. As discussed in Eddy et al.,^26^ the study of kidney diseases presents a unique opportunity to leverage multi-omics data integration, as the necessary biosamples (i.e., kidney biopsies), which are valuable resources for generating genetic, transcriptomic, proteomic, and metabolomic data, are often collected in clinical practice. While single-cell and spatial omics have advanced tissue analysis, they often capture only a single layer of complexity. Integrative multi-omics, which combines various data types, offers a more comprehensive view to enhance our understanding of diseases like DKD and AKI. This integration allows for the identification of noninvasive surrogate markers that reflect cellular processes occurring in the kidney, offering a less invasive method to monitor disease progression and treatment response.

## CHALLENGES FOR MULTI-OMICS DATA INTEGRATION

**Figure 1:**
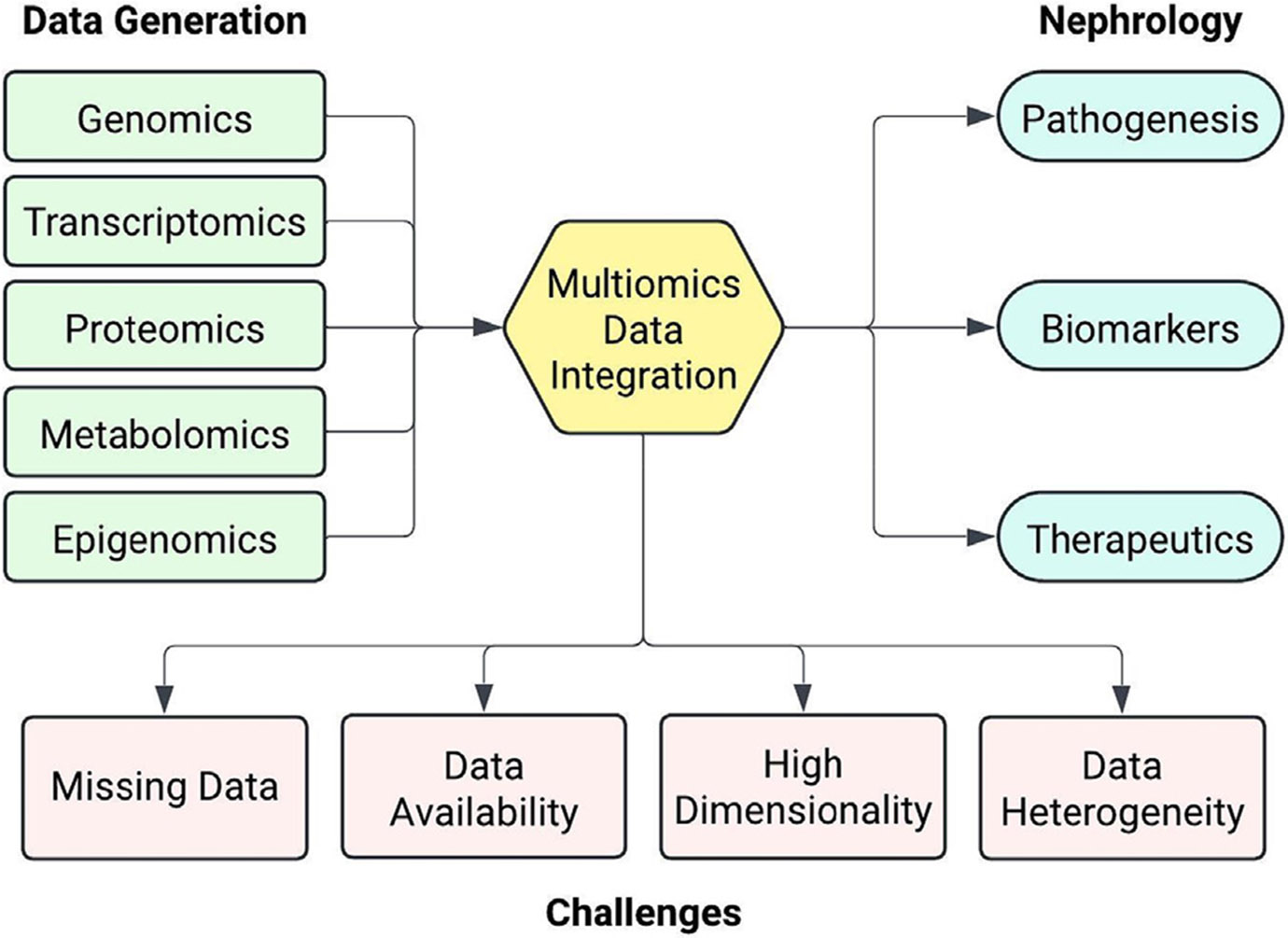
Multi-omics data integration in nephrology research.

Integrating multi-omics datasets poses significant challenges due to their complexity and the inherent variability in biological data (Fig. 1). In the following sections we provide an overview of these challenges.

### Missing Data

Missing data are a common issue in omics studies, whether resulting from technical failures such as poor tissue quality, insufficient sample volume, or measurement system limitations or other factors such as budget restrictions or subject dropout. Missing values in large-scale ‘omics data can significantly hinder downstream analyses,^27^ making the handling of missing data a regular challenge in multi-omics integration and analysis. The variability in the set of observations with missing data and the proportion of missingness across different omics datasets add to this complexity. Readers are referred to more details in the review of Flores et al.^28^

### Data Availability

In the context of nephrology research, a significant challenge is the scarcity of large, diverse datasets, especially in medical imaging-based deep learning.^1, 29^ Moreover, both the initial and continuous model training depend on ongoing data supplementation, validation, and improvement. Therefore, global, secure, and real-time updated resources for the research and clinical communities help improve the availability of larger datasets, which facilitates the development of highly parameterized models, thus offering significant transformative potential. Initiatives like the Kidney Precision Medicine Project,^30-32^ Nephrotic Syndrome Study Network,^33^ Transformative Research in Diabetic Nephropathy,^34^ and Cure Glomeru-lonephropathy^35^ aim to achieve comprehensive characterization of kidney biopsies across various kidney disease subtypes.

### High Dimensionality and Data Heterogeneity

Omics datasets are typically high in dimensionality, with thousands of variables (such as gene expression levels) but relatively few samples. This “curse of dimensionality” can result in overfitting, where models excel on training data but fail on new, unseen data. Different omics layers (e.g., genomics, transcriptomics, proteomics) present data in various formats and scales. Gene expression datasets may have tens of thousands of features, while metabolomics datasets might contain only a few hundred. This heterogeneity further complicates data integration and analysis.

In nephrology, the presence of comorbidities often makes patient cohorts highly heterogeneous, complicating analysis. Therefore, data standardization and harmonization, along with the ability to integrate multimodal datasets, are critical.^36^ Identifying biomarkers for kidney disease involves analyzing subtle patterns across different omics layers, which is complicated by the high dimensionality and variability of the data. Effectively reducing dimensionality and selecting the most influential features are essential for improving model performance and ensuring robust results.

## COMPUTATIONAL METHODS FOR DATA INTEGRATION

Addressing the challenges associated with integrative multi-omics analyses requires a combination of careful data handling, advanced statistical methods, and thoughtful integration strategies to derive meaningful insights from multi-omics data. We outline the types of integration strategies structurally and algorithmically, as well as their application in recent nephrology research to advance clinical insights and potential therapeutic interventions.

### Overview of Integration Strategies

**Figure 2.**
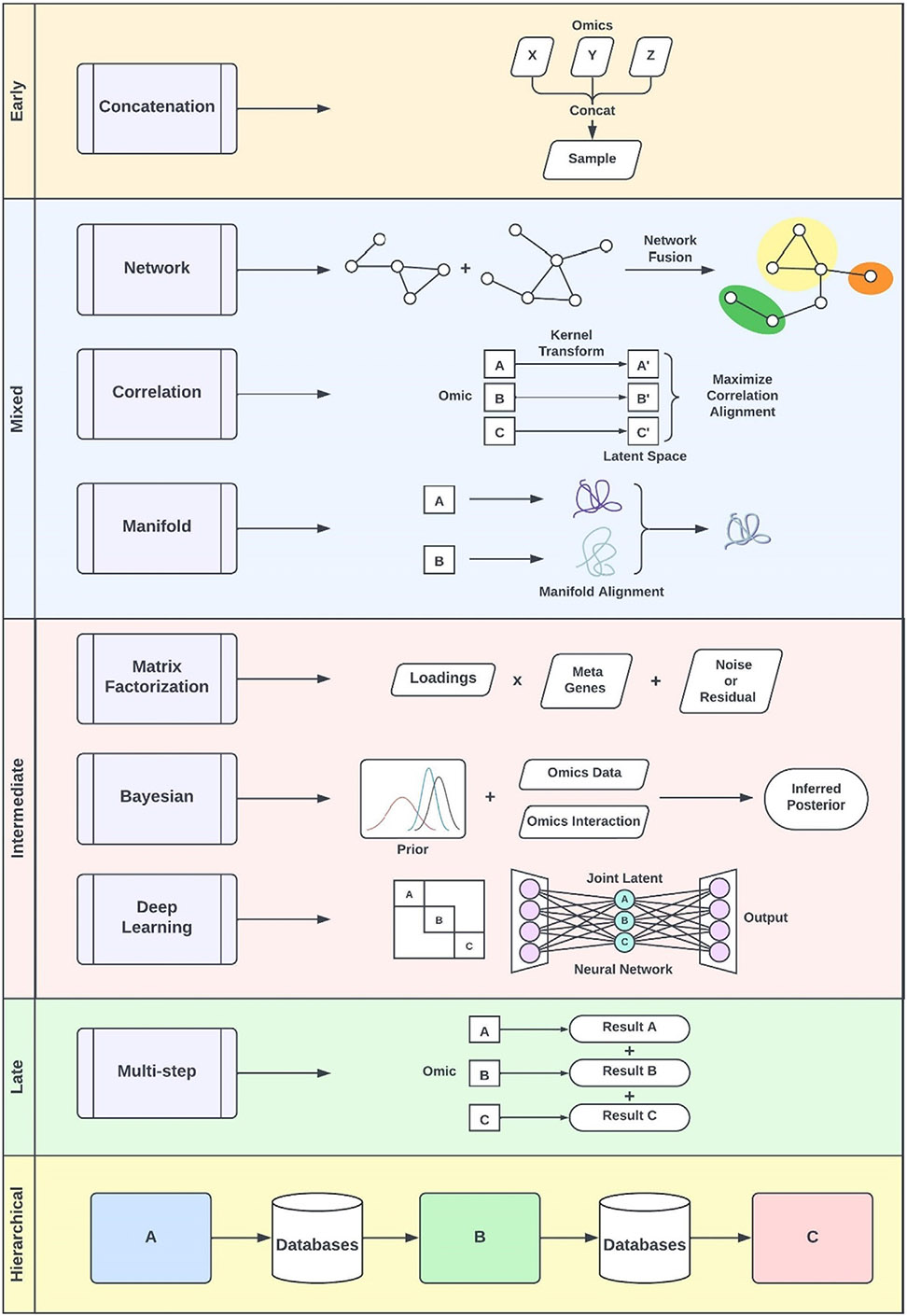
Overview of integration techniques in multi-omics data analysis.

From a structural point of view, most integration tasks for multi-omics measurements can be classified into three categories.^37^ *Horizontal* integration combines data from different samples within the same modality, such as integrating gene expression data from various cohorts for a meta-analysis to increase the statistical power. The major challenge for this type of integration is proper normalization and batch correction.^38^ *Vertical* integration uses common samples or cells as the anchor to integrate simultaneous profiling of multiple modalities. For example, it can integrate simultaneous profiling of scRNA-seq, which measures gene expression, and single-cell assay for transposase-accessible chromatin with sequencing (scATAC-seq), which assesses chromatin accessibility, using the 10x Genomics Multiome platform. Given the mixed embedding space of multimodal features, preserving biological interpretability is challenging in this type of integration. *Diagonal* integration in multi-omics refers to organizing multiple omics datasets into a block-diagonal matrix, where each omics dataset occupies a separate block along the diagonal, allowing for independent but aligned analysis of each omics layer within a unified framework. This diagonal approach presents the greatest challenge, as shared cells or omics features to serve as anchor points for integration are lacking. This approach typically depends on identifying strong correlations among low-dimensional manifolds across different data modalities.^39^ From an algorithmic perspective, integration techniques are traditionally classified into three major levels: early, middle, and late integration, also referred to as concatenation-based, transformation-based, and model-based approaches, respectively.^40^ Furthermore, Picard et al. expanded on this framework by introducing additional categories, specifically early, intermediate, mixed, late, and hierarchical integration.^2^ We illustrate these techniques in Figure 2.

Early integration directly concatenates multiple omics modalities, enabling simultaneous analysis while preserving interactions across different layers.^41^ However, this method is challenged by the inherent heterogeneity of input data, which can vary in distribution, noise levels, and missingness, potentially leading to biased results. Moreover, the high dimensionality relative to the sample size increases the risk of overfitting. *Mixed* integration addresses some of these challenges by first extracting latent representations from each high-dimensional omics layer, before combining them for downstream analysis.^2,42^ This approach reduces dimensionality and noise. However, since each omics layer is transformed independently, there is a risk of losing valuable interplay and correlations between modalities. *Intermediate* integration differs from mixed integration in that it does not require prior concatenation or independent transformation of datasets.^2^ Instead, it seeks to identify a common latent space that all omics data share, such as through nonnegative matrix factorization (NMF).^43^ This approach assumes that multi-omics data converge in a shared latent representation, thereby considering interactions and correlations. However, it often relies on unsupervised methods, which makes it difficult to incorporate prior biological knowledge into the analysis.

*Late* integration is a straightforward approach involving the combination of outputs from individual analyses of each omics layer. For example, Chen et al.^44^ used late integration to combine data from various sources, including epigenome-wide DNA methylation from blood cells, genetic variants, circulating proteins, and microRNAs (miRNAs). They identified specific DNA sites (CpG loci) associated with kidney failure risk in individuals with type 1 diabetes and DKD. By analyzing these sites along plasma proteins and miRNAs, they performed mediation analyses to determine how proteins and miRNAs affect the link between DNA methylation and kidney failure risk. While simple, late integration is limited by its inability to capture shared knowledge across omics layers, thereby undermining the holistic nature of multi-omics analysis.^2^

*Hierarchical* integration adopts a sequential process that incorporates prior biological knowledge, such as pathway and regulatory relationships among omics layers.^45^ Although this method can be highly specific and informative for certain types of data, it often requires extensive external biological knowledge, making it less robust and versatile in handling diverse data types. Methods like Mergeomics^46^ exemplify hierarchical integration. Mergeomics leverages multiple biological databases, including over 25 different pathways and network databases, to identify regulatory pathways using prior biological knowledge. In a study on cisplatin-induced AKI by Deng et al.,^47^ Mergeomics employed marker set enrichment analysis and weighted key driver analysis to integrate multi-omics data and pinpoint key molecular targets. This approach identified tumor-associated calcium signal transducer 2 (*Tacstd2*) as a key driver in the ferroptosis network associated with cisplatin-induced AKI. Functional analyses, including gene ontology and Kyoto encyclopedia of genes and genomes (KEGG), along with network visualization using Cytoscape, provided further insights into *Tacstd2*’s role and its interactions with other genes.

### Description of Data Integration Methods

Herein, we organized the state-of-the-art data integration methods that can be applied to bulk, single-cell, and spatial data, which have been developed to address the aforementioned challenges (Tables 1-3).

### Integration Methods for Bulk Omics Data

Bulk omics integration methods are developed to address problems, mainly focusing on disease subtyping, survival prediction of subtypes, biomarker discovery, and biological insights using large-scale datasets such as gene expression, DNA methylation, CNVs, and mutations.^48^ Bulk omics inputs are usually composed of matrices with rows representing features (gene, CpG, protein, drug response, etc.) and columns representing bulk tissue samples or patients. Huang et al. classified joint analysis of bulk multi-omics data into various categories, including Bayesian, network-based, matrix factorization–based, and multi-step correlation–based methods, involving mix and intermediate integration approaches as mentioned above.^49^

Bayesian methods such as Bayesian consensus clustering (BCC)^98^ and patient-specific data fusion (PSDF)^50^ use the Dirichlet process foundation for clustering and feature selection. MOFA uses a probabilistic Bayesian model supporting a combination of different noise models.^51^ For instance, Clos-Garcia et al. found distinct gut microbiome and blood metabolome signatures in individuals with type 1 diabetes and progressive kidney disease, stratified by albuminuria levels, by integrating multi-omics data using methods like shotgun sequencing, metabolomics profiling, and the MOFA tool for multi-omics analysis.^52^

Network-based methods such as Similarity Network Fusion (SNF)^53^ and Neighborhood-based multi-omics clustering (NEMO)^54^ handle data heterogeneity by constructing and fusing omic-specific similarity matrices, enabling robust subgroup identification across samples.

For example, gene expression and DNA methylation data were integrated using SNF in a study^55^ to classify kidney renal clear cell carcinoma (KIRC) into three stages. By combining these data types, a fused network was created, which improved cancer stage prediction compared to using either data type alone. The fused network’s structure captured patient relationships more effectively, allowing for more accurate classification. This approach demonstrated the power of multi-omics data integration for better disease diagnosis and classification of KIRC.

Matrix factorization-based methods such as Integrative NMF (intNMF),^56^ iCluster+,^57^ and JIVE ^58^ project the data into lower-rank matrices including metagenes (combination of original genes) and loadings (coefficient matrix), facilitating the identification of shared biological patterns while reducing noise. A recent study^59^ integrated data on aneuploidy, DNA hypermethylation, mRNA, and miRNA expression, identifying 28 distinct molecular subtypes, with clustering patterns reflecting histology and tissue type. Using the iCluster algorithm, three kidney cancer subtypes were identified: a pan-kidney cluster, an epithelial-mesenchymal transition cluster, and a heterogeneous group consisting of clear cell, papillary, and chromophobe renal cell carcinomas (RCC). The pan-kidney cluster showed high hypoxia signaling and active immune-related pathways, while chromophobe RCC co-clustered with adrenal cortical carcinoma, lacking hypoxic and immune signals. This analysis enhances the understanding of kidney cancer’s molecular landscape.

On the other hand, correlation-based methods such as mixOmics^60^ implement both canonical correlation analysis and partial least squares to maximize the covariance between different data modalities and mitigate sensitivity to outliers. This analysis is exemplified in a study on kidney transplant rejection^61^ applying various techniques, including principal component analysis (PCA), integrative PCA, sparse partial least squares discriminant analysis, and sparse generalized canonical correlation analysis (currently accessible in mixOmics). The study integrated genomics and proteomics data from patients with acute rejection and nonrejecting controls, identifying key gene and protein signatures associated with immune responses. Although most integration methods require paired samples across multiple modalities to proceed, methods like JIVE,^58^ multi-omics factor analysis (MOFA),^51^ and NEMO^54^ can be applied to partially paired samples to solve the missing data dilemma described earlier.

Last but not least, the multistep integration combines data from various omics layers to extract meaningful insights in a structured manner and then integrates them to capture shared patterns and interactions, focusing on the consensus and association between omics.^40^ Haug et al.^62^ used the multistep approach to explore the renal medulla, creating a detailed genomic reference for adult kidneys. By integrating individual results from bulk RNA-seq, ATAC-seq, and chromatin conformation with spatial transcriptomics and immunohistochemistry, they identified 31 high-confidence marker genes and provided clear distinctions between medullary and cortical tissues. Additionally, the study reclassified genotype-tissue expression project (GTEx) kidney samples into medullary or cortical categories using PCA. This combined GTEx data with the study’s RNA-seq data, allowing for differential analysis across a broader dataset. It also highlighted medullary-specific enhancer regions and potential regulatory roles of genes like claudin 14 (*CLDN14*) and Wnt family member 7B (*WNT7B*).

### Integration Methods for Single-Cell Omics Data

Unlike bulk omics data, single-cell omics datasets provide information at a single-cell resolution, enabling more precise understanding of various aspects, including cell type classification, detailed modeling of gene regulatory networks, and the biological process under study.^63^ In single-cell multi-omics analysis, the objective is to integrate diverse modalities into a shared latent space where the data can be jointly analyzed to foster downstream analyses (cell lineage tracing, tissue atlas, assessing microenvironment, etc.).^64^ These integration methods can be broadly categorized into paired and unpaired strategies, the latter of which, unpaired methods, is also called *alignment*.^65^

In the case of paired data where multiple omics layers are collected simultaneously from the same cell, Stanojevic et al. summarized the common integration method as matrix factorization–based, neural network–based, and network-based approaches.^65^ Matrix factorization–based methods like MOFA+^66^ employ hierarchical generative modeling and variational inference to produce unified latent representations. Deep learning–based methods like MultiVI^67^ and Cobolt^68^ similarly use variational autoencoders that model each omic layer with its own distinct distribution. Network-based methods like Signac^69^ focus specifically on two omics, such as scRNA-seq and scATAC-seq, leveraging latent semantic indexing (LSI) to treat chromatin accessibility data in a way analogous to natural language processing.^70^

In a study of DKD,^71^ researchers used Signac to analyze single-nucleus RNA-seq (snRNA-seq) and single-nucleus ATAC-seq (snATAC-seq) data from kidney cortex samples from patients with and without type 2 diabetes, to integrate chromatin accessibility with gene expression profiles. They identified accessible chromatin regions across various cells, with a focus on proximal convoluted tubules cells. Signac enabled batch effect correction and doublet removal, facilitating the identification of chromatin accessibility linked to cell type–specific markers. This approach uncovered differential chromatin features and transcription factor binding sites associated with glucose metabolism and glucocorticoid signaling.

A study investigating the role of mineralocorticoid excess in hypertension and kidney disease^72^ employed Signac for scATAC-seq analysis identifying chromatin accessibility changes in principal and connecting tubule cells and highlighting the protective effects of antihypertensive therapies in a rat model. Signac was also used to explore how a Wilms tumor–associated mutation in the histone acetylation reader eleven-nineteen-leukemia (ENL) disrupts kidney differentiation in mice,^73^ revealing alterations in the gene regulatory landscape that promote nephron progenitor commitment while hindering their maturation, ultimately leading to severe developmental defects, which could be reversed by a small molecule inhibitor targeting mutant ENL.

In contrast, unpaired methods are designed to integrate omics data obtained from different experiments and cell types without requiring direct correspondence between datasets. A common approach in these methods is to map the data into a shared co-embedded space or a nonlinear manifold to identify common patterns.^65^ LIGER^74^ uses iNMF to align and analyze heterogeneous datasets. GLUE^75^ applies graph-linked variational autoencoders and adversarial alignment to learn unified feature representations while simultaneously reconstructing omics layers from unaligned datasets. Seurat^76^ integrates data using CCA combined with mutual nearest neighbor identification to anchor features. One study on autosomal dominant polycystic kidney disease (ADPKD)^77^ analyzed single-nucleus multi-omics data from a mouse model, integrating transcriptomic and epigenetic datasets while using Harmony for batch effect correction. Uniform manifold approximation and projection (UMAP) and a weighted nearest neighbor graph were employed for dimensional reduction and integration of snRNA-seq and snATAC-seq data. Seurat’s Find-Markers function identified differentially expressed genes and accessible chromatin regions, revealing cell type–specific responses to polycystic kidney disease (*Pkd1*) deletion. In another related study,^78^ snRNA-seq data from ADPKD and healthy human kidney samples were analyzed. Harmony was again used for batch effect correction, with UMAP for dimensionality reduction and the Louvain algorithm for clustering. This study identified key signaling pathways, particularly in proximal tubular cells, and highlighted G protein–coupled receptor class C group 5 member A (*GPRC5A*) as a marker for cyst-lining collecting duct cells. Both studies by Humphreys et al.^77,78^ utilized Seurat for single-cell-level integration and analysis, providing insight into the early molecular deregulation in a mouse model of PKD and human ADPKD kidney pathology.

### Spatial Omics Integration Methods

Spatial omics integration is crucial for capturing tissue architecture and deciphering intricate cell–cell interactions with spatial and temporal precision. However, spatial assays often face inherent trade-offs between resolution and the breadth of molecular features they can access. For instance, platforms like 10x Genomics’ Visium offer spot-level resolution, typically capturing around 10 cells per spot while offering comprehensive transcriptomic profiling. On the other hand, matrix-assisted laser desorption/ionization mass spectrometry imaging (MALDI-MSI) provides high spatial resolution, enabling visualization of spatial metabolites within tissues.

This integration of datasets is further enhanced when combining transcriptomic, chromatin accessibility, and metabolic features that overlap with single-cell data. Computational tools like LIGER and Seurat can leverage these shared features for robust alignment and joint analysis. For example, in a recent study, Humphreys and colleagues used the 10X Visium platform to spatially map transcriptomic data in human kidney tissue. They integrated these data with histopathological analysis by performing morphology-based clustering. Seurat was then used to normalize the transcriptomic data, perform clustering, and reduce dimensionality, allowing morphology-based clusters integration with gene expression profiles. This integration helped align molecular signatures with specific tissue morphologies, enhancing the interpretation of kidney lesions associated with CKD.^79^

Rabelink and colleagues employed MALDI-MSI to map lipidome and metabolome across renal structures in the developing human fetal kidney. They performed post-MALDI immunofluorescence staining for coregistration with the MALDI-MSI data, which enhanced the identification of distinct renal cell types. Using Seurat, they integrated scRNA-seq data with spatial metabolomics and isotopic tracing. This multi-omics approach linked specific metabolic signatures to distinct renal cell types, revealing a shift in substrate utilization from glycolysis to fatty acid oxidation during proximal tubules differentiation.^80^ Similarly, in a related study investigating metabolic changes in kidney tissue after ischemia-reperfusion injury in mice, the team also integrated MALDI-MSI with immunofluorescence data. They conducted MSI measurements on kidney tissue sections at various time points and with different isotope-enriched nutrients. To capture dynamic metabolic changes at pixel level while minimizing batch effects, they employed a two-step process from the Seurat package. First, anchors were identified between datasets using single-pixel lipid profiles; then, they transferred the abundance of isotope-enriched metabolites into the control dataset via k-nearest neighbors (k-NN) analysis. This process established comprehensive isotope-labeling information, enabling calculation of dynamic metabolic changes and the generation of pseudoimages that visualized the *in situ* heterogeneity of metabolic dynamics in ischemic injury.^81^

Despite the advantages of spatial assays, challenges remain in balancing resolution with the breadth of omics features. Technologies such as 10x Xenium, NanoString, CosMx, and MERFISH offer single-cell resolution but are limited in transcriptomic coverage, typically measuring a few hundred to a few thousand genes. This reduced depth presents challenges for detailed biological analysis. To address this, methods like ENVI,^82^ Tangram,^83^ stPlus,^84^ and CellTrek^85^ have been developed to impute missing gene expression in spatial data by leveraging scRNA-seq information. These tools integrate reconstructed expression data with spatial datasets, facilitating the alignment and joint analysis of single-cell and spatial omics data. Instead of imputing gene expression, methods like CellTrek use a spatial mapping algorithm to project cells onto spatial transcriptomic coordinates and employ random forest and mutual nearest neighbor to impute the spatial coordinates of single-cell data. For example, in a recent study addressing the kidney’s complex three-dimensional cellular structure, Susztak et al.^86^ generated high-quality datasets from numerous samples of healthy, diabetic, and hypertensive kidney tissues. They utilized single-cell, single-nucleus, and spatial transcriptomics data. Using Signac, they analyzed snRNA-seq and snATAC-seq data to identify cell types and chromatin accessibility. These cell types were mapped to spatial transcriptomic data using Cell2location, a Bayesian model that enables the integration of single-cell and spatial transcriptomics with higher sensitivity and resolution.^87^ CellTrek was employed to impute precise cell locations at near single-cell resolution. This integration revealed distinct kidney microenvironments, such as glomerular, immune, tubular, and fibrotic regions, providing new insights into the spatial organization of healthy and diseased kidney tissues.

Other spatial omics tools, including SpaGCN^88^ and GraphST,^89^ integrate spatial information and single-omics modalities by applying graph neural networks (GNNs) to capture spatial domains, enhancing the interpretation of tissue architecture. They can identify spatial domains that preserve temporal and functional information. However, these methods only support unimodal input, and feature concatenation is needed if applied on the multimodality scale assuming the same weight.

Unlike the previously mentioned methods, which focus primarily on aligning spatial transcriptomics with single-omics or single-modality data, SpatialGlue^90^ is currently the only method designed for spatial multi-omics data integration using a GNN framework with the dual-attention mechanism. SpatialGlue first uses k-NN to construct a spatial neighborhood graph and then applies a GNN with an attention mechanism to learn modality-specific representations. This allows for the detailed analysis of complex tissue structures and cell interactions across different molecular modalities.

## FUTURE PERSPECTIVE AND PREDICTIONS IN THE FIELD

The field of nephrology stands on the cusp of a transformative era driven by the integration of high-dimensional omics data across different measurement scales. The advancements in multi-omics technologies, as discussed in this review, have already begun to unravel the complex molecular underpinnings of kidney diseases. We are optimistic that the future holds even greater promise as these technologies continue to evolve and become more sophisticated. These developments will be important to pursue to implement precision nephrology.

### Enhanced Data Integration and Interpretation

Future efforts in nephrology will likely focus on enhancing the integration of multi-omics data across different resolutions and modalities. The development of more advanced computational algorithms that can handle the increasing complexity and heterogeneity of these datasets will be crucial for this task. These algorithms will need to seamlessly integrate genomics, transcriptomics, proteomics, metabolomics, and spatial data to provide a holistic understanding of kidney disease mechanisms.

### Integration With Artificial Intelligence and Machine Learning

The future of multi-omics integration can be closely tied to and benefit from the most recent advancements in AI and ML. These technologies will play a pivotal role in identifying patterns and correlations within complex omics datasets that may not be apparent through traditional analysis methods. AI-driven models could predict disease progression, identify novel therapeutic targets, and even suggest personalized treatment plans based on a patient’s unique omics profile. Importantly, some AI and ML models can be adapted to increasingly accessible large language models^91^ and foundation models,^92^ making model retraining more efficient.

### Broader Data Accessibility and Global Collaboration

The accuracy of the multi-omics integration and AI models in nephrology will undoubtedly depend on broader accessibility to these technologies and global collaboration. Developing open-access data repositories and opensource code/packages will ensure that researchers and clinicians worldwide have the necessary tools and datasets to advance the field. International consortiums and cross-disciplinary collaborations will foster the sharing of knowledge and resources, accelerating the pace of discovery and translating research findings into clinical practice more rapidly.

### Real-Time Personalized Medicine

The ultimate goal of multi-omics integration in nephrology is the realization of real-time, personalized medicine. As these technologies mature, we anticipate the emergence of clinical tools that can integrate patient-specific omics data in real time, enabling personalized treatment strategies. This will be particularly transformative for diseases like CKD and AKI, where early detection and individualized treatment can significantly improve outcomes.

### Crosstalk Between Nephrology and Other Diseases

The kidneys play a critical role in maintaining overall health by filtering waste, balancing fluids, and regulating blood pressure, among other functions. Disruptions in kidney function can have widespread effects on other systems, and vice versa. There have been known connections between kidney diseases and other diseases including cardiovascular diseases, lung diseases, cancers, and neurodegenerative diseases.^93-96,100^ Kidney diseases could also be complications of other diseases, such as preeclampsia during pregnancy.^97^ It is expected that multi-organ or system-level research using tools such as multi-omics will reveal new holistic insights for the long-term management of patients to improve their life span and quality of life.

## Figures and Tables

**Table 1.** Integration Methods for Bulk Omics Data

Method	Field of Application	Algorithm	Sample Requirement	Supported Input Data	Reference
BCC	Subtyping	Bayesian consensus clustering	Paired	Omics data	, ^98^
iClusterPlus	Subtyping, Biomarker	Gaussian latent variable model	Paired	CNV, Omics data	^57^
intNMF	Subtyping	Non-negative matrix factorization	Paired	CNV, Omics data	^56^
JIVE	Subtyping, Insights	Joint and individual variation	Partially paired	Omics data	^58^
mixOmics	Subtyping, Biomarker	Canonical correlation, Partial least squares	Paired	Omics data	^60^
MOFA	Subtyping, Biomarker	Factor analysis	Partially paired	Mutation, Omics data, Drug Response	^51^
NEMO	Subtyping	Similarity	Partially paired	Omics data	^54^
PSDF	Subtyping	Bayesian	Paired	Omics data	^50^
SNF	Subtyping	Similarity network fusion	Paired	Omics data, Discrete data	^53^

**Table 2.** Integration Methods for Single-Cell Omics Data

Method	Field of Application	Algorithm	Sample Requirement	Supported Input Data	Reference
Cobolt	Joint latent	Hierarchical generative model	Paired	scRNA-seq, scATAC-seq	^68^
GLUE	Joint latent	Graph-linked unified embedding	Unpaired	scRNA-seq, scATAC-seq, scmC-seq	^75^
Signac	Joint latent	Reciprocal LSI projection	Paired	scRNA-seq, scATAC-seq	^69^
UnionCom	Joint latent	Manifold alignment	Unpaired	scRNA-seq, scATAC-seq, scDNAm	^99^
MOFA+	Joint latent	Stochastic variational inference	Paired	scRNA-seq, scATAC-seq, Methyl	^66^
MultiVI	Joint latent	Deep generative model	Paired	scRNA-seq, scATAC-seq, Protein	^67^
LIGER	Joint latent	Matrix factorization	Unpaired	scRNA-seq, scATAC-seq, snDNAm	^74^
Seurat	Joint latent	Weighted nearest neighbor	Unpaired	scRNA-seq, scATAC-seq, Protein	^76^

**Table 3.** Integration Methods for Spatial Omics Data

Method	Field of Application	Algorithm	Sample Requirement	Supported Input Data	Reference
LIGER	Joint latent	Matrix factorization	Unpaired	Single-cell data, Spatial data	^74^
Seurat	Joint latent	Weighted nearest neighbor	Unpaired	Single-cell data, Spatial data	^76^
ENVI	Mapping	Variational inference	Unpaired	scRNA-seq, Spatial transcriptomics	^82^
Tangram	Mapping	Nonconvex optimization	Paired	scRNA-seq, Spatial transcriptomics	^83^
stPlus	Mapping	Autoencoder, Weighted k-NN	Unpaired	scRNA-seq, Spatial transcriptomics	^84^
CellTrek	Mapping	Random forest, Mutual nearest neighbors	Unpaired	scRNA-seq, Spatial transcriptomics	^85^
SpaGCN	Joint latent, Spatial domain	Graph convolutional network	Paired	Spatial transcriptomics with histology	^88^
GraphST	Alignment, Spatial domain	Graph self-supervised contrastive learning	Paired	Spatial transcriptomics with histology	^89^
SpatialGlue	Joint latent, Spatial domain	Graph neural network with dual attention	Partially paired	Spatial epigenome, transcriptome, proteome	^90^
